# BCAS2 Enhances Carcinogenic Effects of Estrogen Receptor Alpha in Breast Cancer Cells

**DOI:** 10.3390/ijms20040966

**Published:** 2019-02-22

**Authors:** Ángel Salmerón-Hernández, María Yamilet Noriega-Reyes, Albert Jordan, Noemi Baranda-Avila, Elizabeth Langley

**Affiliations:** 1 Departamento de Investigación Básica, Instituto Nacional de Cancerología, Av. San Fernando No. 22, Col. Sección XVI, 14080 Mexico City, Mexico; angel.salher@yahoo.com.mx (A.S.-H.); yamilet_noriega@hotmail.com (M.Y.N.-R.); mimi19481@yahoo.com.mx (N.B.-A.); 2 Programa de Doctorado en Ciencias Biomédicas, Universidad Nacional Autónoma de México, 04510 Mexico City, Mexico; 3 Institut de Biología Molecular de Barcelona (IBMB-CSIC) Parc Científic de Barcelona, Barcelona, 08028 Cataluña, Spain; ajvbmc@ibmb.csic.es

**Keywords:** estrogen receptor, breast cancer, BCAS2, coactivators

## Abstract

Estrogen receptor alpha (ERα) has an established role in breast cancer biology. Transcriptional activation by ERα is a multistep process modulated by coactivator and corepressor proteins. Breast Cancer Amplified Sequence 2 (BCAS2), is a poorly studied ERα coactivator. In this work, we characterize some of the mechanisms through which this protein increases ERα activity and how this promotes carcinogenic processes in breast cancer cells. Using protein-protein interaction and luciferase assays we show that BCAS2 interacts with ERα both in vitro and in vivo and upregulates transcriptional activation of ERα directly through its N-terminal region (AF-1) and indirectly through its C-terminal (AF-2) region, acting in concert with AF-2 interacting coactivators. Elevated expression of BCAS2 positively affects proliferation, clonogenicity and migration of breast cancer cells and directly activates ERα regulated genes which have been shown to play a role in tumor growth and progression. Finally, we used signal transduction pathway inhibitors to elucidate how BCAS2 is regulated in these cells and observed that BCAS2 is preferentially regulated by the PI3K/AKT signaling pathway. BCAS2 is an AF-1 coactivator of ERα whose overexpression promotes carcinogenic processes, suggesting an important role in the development of estrogen-receptor positive breast cancer.

## 1. Introduction

ERα is the primary therapeutic target in breast cancer and is expressed in close to 70% of cases. These tumors can respond to endocrine therapy and be growth inhibited by pharmacologic blockade of estrogen production or ER. The selective ER modulator, Tamoxifen (TAM), is the most widely used hormonal therapy. However, prolonged treatment often derives in endocrine resistance and tumor recurrence [1], through mechanisms which have not been completely elucidated. Some of these mechanisms include ERα loss or mutations, changes in coregulatory proteins, increased cross-talk with other signal transduction pathways resulting in aberrant or ligand independent activation of ER or its coregulators and alternative downstream signaling pathways activating different gene expression programs [2,3,4,5,6]. The transcriptional activity of ERα is dependent on several coregulatory proteins called coactivators and corepressors [7,8]. Coactivators do not usually bind DNA directly but are recruited to target gene promoters through protein-protein interactions with nuclear receptors (NRs). Thus, Ligand binding promotes the formation of multiprotein complexes containing the nuclear receptor (NR), coactivators and basal transcription factors acting in concert to activate transcription [8]. Structural analysis of coactivators has identified a five amino acid (aa) motif, L*XX*LL (where *X* is any amino acid), which is sufficient to mediate coregulator binding to the liganded NRs at their AF-2 domain [9]. However, a number of coactivators have recently been discovered that bind to the N-terminus of NRs and activate the AF-1 transcriptional activation function. In general, coactivators increase transcriptional activity through chromatin remodeling, histone acetylation or methylation, as well as recruitment of other coregulators and of the basal transcriptional machinery [10,11]. In contrast, corepressors associate with histone deacetylases to repress transcription and promote a closed chromatin configuration [12]. Besides modulating chromatin structure to activate or repress transcription, coactivators and corepressors can have many other functions including control of splicing and protein degradation through ubiquitination. [13]. Additionally, expression of different coregulators has been implicated in differential tissue and cell type-specific responses to various hormones; however, more research is required to fully understand these mechanisms.

Using a yeast two-hybrid assay, we detected BCAS2 as an ERα binding protein, interacting with its N-terminal domain. BCAS2 was previously determined to be a coactivator protein that increases ERα transcriptional activity through its AF-2 domain [14] and has been found to associate with the tumor suppressor p53 protein [15]. In this work, we identified BCAS2 as a protein that interacts with ERα both in vitro and in vivo and regulates the transcriptional activation of ERα through its N-terminal region (AF-1) and indirectly via the C-terminal (AF-2) region. The enhanced expression of BCAS2 in human mammary cancer cell lines increases their proliferation, migration and colony formation. Furthermore, it regulates the expression of genes that have a role in breast cancer tumorigenesis. This suggests that BCAS2 regulates AF-1 activity on the ERα N-terminus and may play a role in regulating estrogen dependent growth in breast cancer. 

## 2. Results

### 2.1. BCAS2 Interacts Directly with the N-Terminal Region of ERα

**Figure 1:**
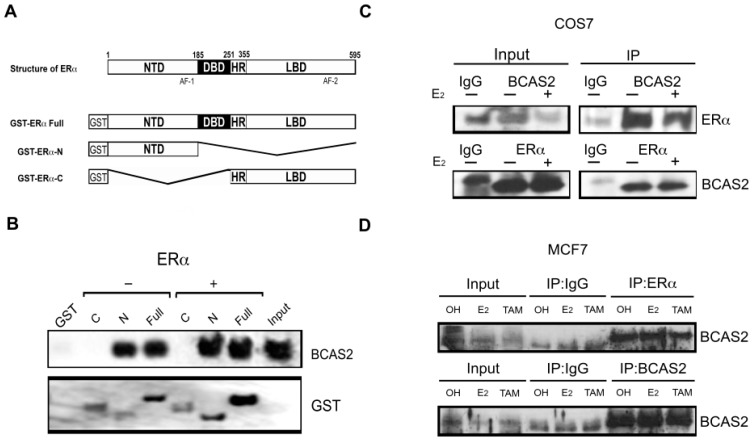
BCAS2 interacts with ERα in vivo and in vitro. (**A**) Structure of ERα and its N and C domains used for Glutathione sepharose affinity matrix assays. NTD, amino terminal domain; DBD, DNA binding domain; HR, hinge region; LBD, ligand binding domain. (**B**) GST pull-down assays of biotin labeled in vitro translated BCAS2 with GST alone, GST-ERα-Full (full-length aa 1-595), GST-ERα-N (aa 1-180) GST-ERα-C (aa 264-595). Western blot analysis was carried out using anti-biotin or anti-GST antibodies. Binding was assayed in the presence (+) or absence (−) of 100 nM E2. (**C**) Coimmunoprecipitation of ERα and BCAS2. COS7 cells were transfected with plasmids expressing ERα and BCAS2 in the presence (+) or absence (−) of 10 nM E2. Immunoprecipitation of whole cell protein extracts was carried out with antibodies against BCAS2 or ERα and IgG as negative control. Shown are immunoblots probed with antibodies against both proteins. (**D**) Coimmunoprecipitation assays in MCF7 cells in the presence of ethanol (OH), E_2_ (10 nM) or TAM (100 nM) showing interaction in the presence or absence of ligand. Full blots can be found in supplementary Figure S1.

Using the yeast two-hybrid system to identify proteins that interact with the N-terminal domain of ERα (aa 1-180), we obtained several sequences that encode for proteins that interact with this region, including BCAS2. To verify this interaction and the involvement of the different domains in BCAS2 binding, we performed pull-down assays in vitro using full-length ERα (Full) as well as its N- and C-terminal domains separately, fused to GST (Figure 1A). Assays were carried out in the presence and absence of E2 and interaction was tested with in vitro labeled BCAS2. We observed that BCAS2 interacts with full-length ERα, both in the presence and absence of E2 and that this interaction takes place via the N-terminal domain of ERα and not through its C-terminal domain, even in the presence of ligand (Figure 1B). Additionally, we determined interaction with ERβ and also found that BCAS2 interacts via its N-terminal region (data not shown). This supports our two-hybrid interaction assay but contrasts previous findings where BCAS2 was found to activate ERα only through its C-terminal domain [14].

Coimmunoprecipitation assays in both, transfected monkey kidney COS7 cells and breast cancer MCF7 cells, confirmed interaction between BCAS2 and ERα (Figure 1C,D) in the absence and presence of E2. Additionally, TAM treatment in MCF7 cells did not abrogate BCAS2 binding to ERα. Thus, indicating a fruitful in vivo interaction between these proteins and suggesting that BCAS2 plays a role in directly regulating ERα activity.

### 2.2. BCAS2 Enhances ERα Transcriptional Activity 

**Figure 2:**
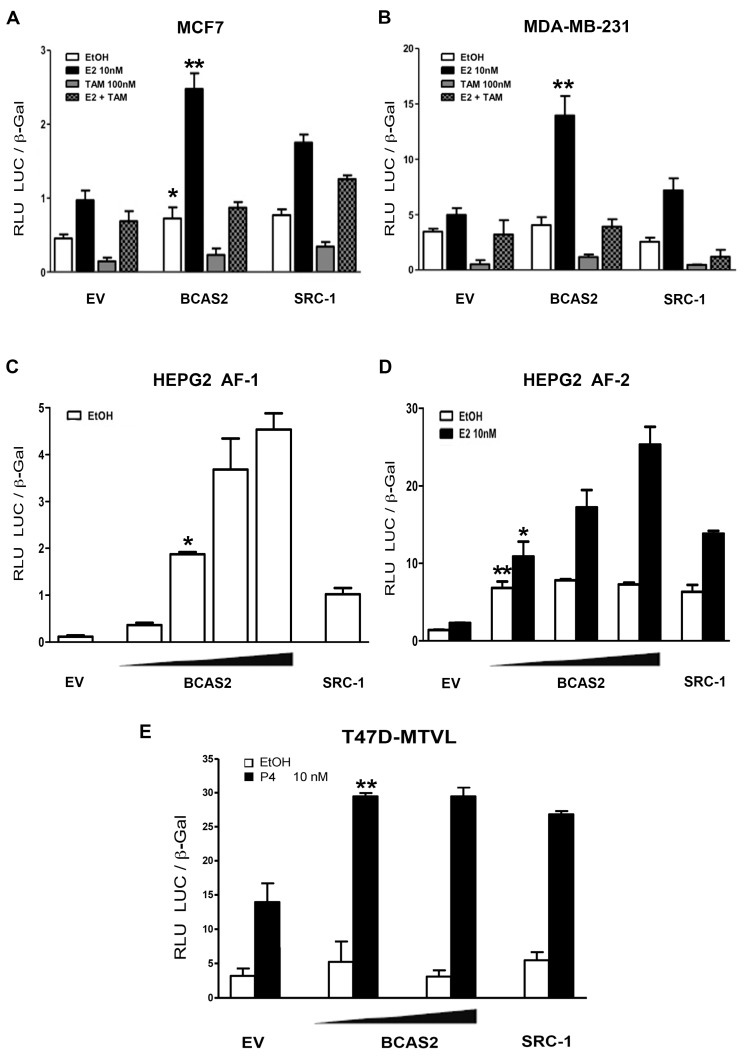
BCAS2 enhances ERα transcriptional activity. Luciferase activity induced by the overexpression of BCAS2 in the presence of ethanol (EtOH), 10 nM E2 or 100 nM TAM (**A**,**B**). MCF7 cells were transiently transfected with an ERE-luciferase reporter vector (200 ng) and BCAS2 (100 ng). Empty pcDNA3.1 vector (EV) (100 ng) and SRC-1 (100 ng) were transfected as negative and positive controls, respectively. (**B**) MDA-MB-231 cells were transfected with the same plasmids as well as 50 ng of ERα. (**C**,**D**) BCAS2 enhances AF-1 (aa 1-282) and AF-2 (aa 144-595) transcriptional activation functions, independently. Each plasmid was transfected into HEPG2 cells along with empty vector (EV) or increasing concentrations of BCAS2 (50-200 ng), ERE-luc and β-galactosidase in the presence or absence of 10 nM E2. Transfected SRC-1 coactivator is used as positive control for transcriptional activation. (**E**) BCAS2 activates PR transcriptional activity. Increasing concentrations of BCAS2 (100 and 200 ng), as well as β-galactosidase vector, were transfected into breast cancer T47D cells stably transfected with a luciferase reporter vector for PR activity and treated in the absence (EtOH) or presence of 10 nM P4. SRC-1 is used as positive control. Relative luciferase activity (RLU LUC/β-gal) is shown as the mean ± SE of a representative assay carried out in triplicate, at least three times. Statistical analysis is carried out by Student´s t-test comparing activation between BCAS2 and empty vector (transfected at the same concentration) in the presence and absence of E2, * *p* < 0.05, ** *p* < 0.001.

It was previously shown that BCAS2 could increase ERα transcriptional activity in CV-1 monkey kidney cells [14]. Thus, we were interested in confirming whether BCAS2 could promote increased ERα transcriptional activity in breast cancer cells. Luciferase reporter gene assays showed that overexpression of BCAS2 is able to enhance ERα transcriptional activity to an extent similar or greater than that found for the SRC-1 coactivator in ER positive (MCF7) (Figure 2A) or ER negative (MDA-MB-231) breast cancer cell lines in the presence of E2 (Figure 2B). Since ERα contains two transcriptional activation functions, which can act independently or cooperatively, we were interested in determining whether BCAS2 could activate both domains. Thus, transcriptional activity assays were performed using the AF-1 and AF-2 domains separately in HEPG2 cells, where it was previously shown that the AF-1 domain can promote independent transcriptional activity [16]. Results show that BCAS2 increases both AF-1 and AF-2 mediated transcriptional activity, in a dose dependent manner (Figure 2C,D). This compares with the activity of SRC-1, which also enhances both transcriptional activation functions [17].

### 2.3. BCAS2 Has a Positive Effect on the Transcriptional Activity of PR

It is known that many coregulators can function on different SHRs. BCAS2 has previously been found to interact with PR in vitro, however, the effect of this interaction was not determined [14]. Therefore, we tested the effect of BCAS2 on the transcriptional activity of PR in a breast cancer cell line (T-47D-MTVL) harboring a PR-dependent luciferase reporter gene [18]. BCAS2 showed a positive effect on PR transcriptional activity in the presence of progesterone (P4) (Figure 2E). Furthermore, AR activity as determined by luciferase reporter assays is also increased by BCAS2 in prostate and breast cancer cells (data not shown and [19]. This suggests that BCAS2 is a coactivator that can be shared between steroid hormone receptors and activates important growth modulators in breast cancer cells. 

### 2.4. BCAS2 Is Recruited to ER Target Gene Promoters and Increases Target Gene Expression

**Figure 3:**
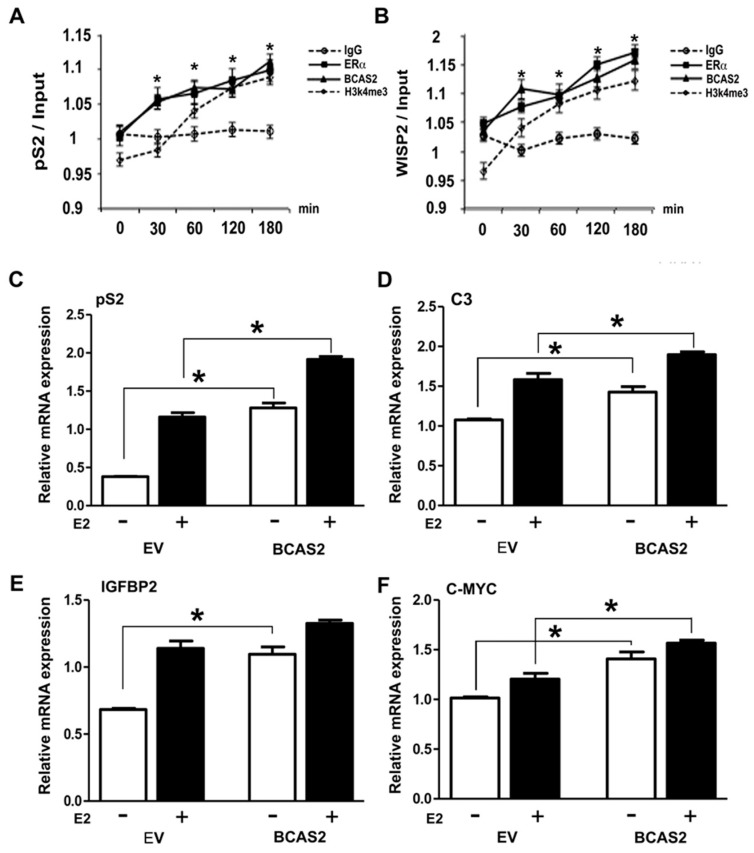
BCAS2 colocalizes with ERα on estrogen regulated promoters upon incubation with E2 and promotes increased estrogen-regulated gene expression. (**A**,**B**) Chromatin immunoprecipitation was performed in MCF7 cells after different times of exposure to E2 (10 nM) treatment (30–180 min). Statistical analysis was carried out comparing ER or BCAS2 with IgG control using by one-way ANOVA, * *p* < 0.001. We carried out qRT-PCR of precipitated DNA showing endogenous ERα and BCAS2 localization at promoter regions of *pS2* and *WISP2* genes. Additionally, Histone 3 methylation (H3k4me3) is shown as a marker for open chromatin. (**C**–**F**) MCF7 cells transfected with pcDNA-BCAS2 or empty vector (EV) and incubated in the presence (+) or absence (−) of 10nM E2 for 24h. RTqPCR was used to determine mRNA expression of *pS2*, *C3*, insulin-like growth factor binding protein 2 (*IGFBP2*) and *C-MYC* and the results were normalized against human GAPDH mRNA amplification. * *p* < 0.05 compared to the EV group at 24 h in the absence (−) or presence of E2, as indicated in the figure.

In order to determine whether BCAS2 is a bona fide coactivator, acting on estrogen responsive promoters, we carried out ChIP assays to determine whether ERα and BCAS2 could be tethered to the pS2 [20] and WISP2 [21], promoters after different times of induction with E2. We found BCAS2 is recruited to these promoters and that its presence on the promoter begins to show in as little as 30 min after exposure to E2 and increases over time (Figure 3A,B). Additionally, we used a marker of open chromatin, H3K4me3, which also shows enrichment after E2 treatment. Thus, we can conclude that BCAS2 is a true coactivator of ERα exerting its effect directly on E2 regulated promoters and that the length of exposure to E2 directly influences this interaction.

We then used RT-PCR to observe transcription of a number of ER regulated genes in MCF7 cells. Overexpression of BCAS2, both in the absence and presence of E2, affects the expression of some genes containing response elements for ER receptors, as shown in Figure 3C–F. We see an increase in mRNA expression for the *pS2*, *C3*, *IGFBP2* and *C-MYC* genes, suggesting BCAS2 can activate a subset of genes depending on the promoter context.

### 2.5. BCAS2 Acts Together with Other Coactivators to Enhance ERα Activity

**Figure 4:**
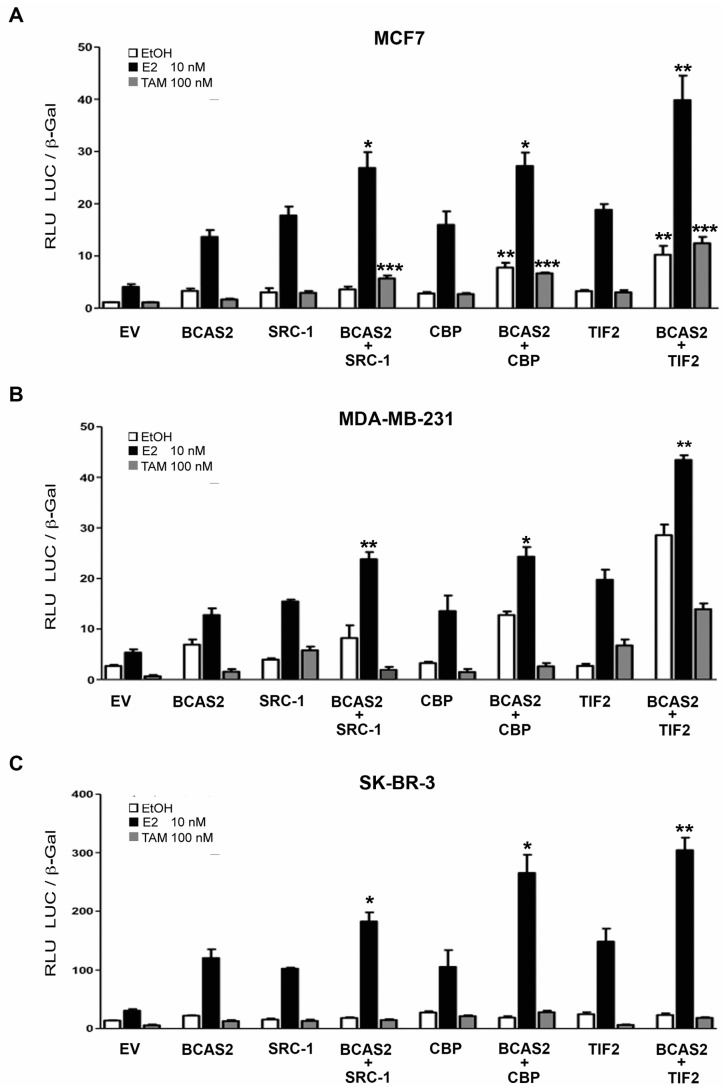
BCAS2 acts together with AF-2 coactivators to increase ERα activity. (**A**) Luciferase assays were carried out with pcDNA-BCAS2 or empty vector (EV), transfected with and without coactivators SRC-1, CBP or TIF2 and treated in the absence (EtOH) or presence of 10 nM of E2 or 100 nM of TAM in MCF7 cells. Similar assays were carried out in MDA-MB-231 (**B**) and SK-BR-3 (**C**) cells with the addition of transfected pSG5-ER Statistical analysis was carried out using one-way ANOVA with Bonferroni posttest; * *p* < 0.01, ** *p* < 0.005, *** *p* < 0.001.

In order to delve further into the molecular mechanisms of the BCAS2 coactivator on ERα transcriptional activation, we evaluated whether BCAS2 was capable of affecting the activity of other ERα coactivators preferentially recruited through the AF-2 domain. We tested SRC-1, TIF2 and CBP. All contain LxxLL motifs through which they bind to AF-2. We tested three different cancer cell lines, MCF7, MDA-MB-231 and SK-BR-3 cells. All of our results show that when BCAS2 is combined with any of the three coactivators in the presence of E2, transcriptional activation is enhanced compared to BCAS2 or the coactivator alone (Figure 4A–C). Interestingly, in MCF7 cells (Figure 4A), the combination of BCAS2 with any of the three AF-2 coactivators, also consistently increases transcription in the presence of TAM, which is not observed in the ER^−^ SK-BR-3 (Figure 4C) or in the MDA-MD-231 cell lines (Figure 4B). This suggests that BCAS2 may play a role in TAM resistance in an ER^+^ cellular environment.

### 2.6. BCAS2 Promotes Cell Proliferation and Colony Formation in Breast Cancer Cells

**Figure 5:**
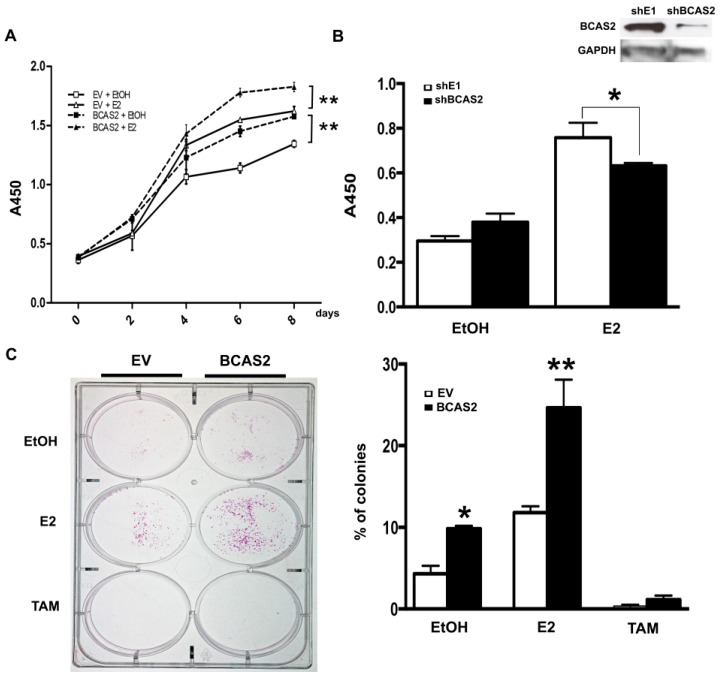
BCAS2 enhances breast cancer cell growth and colony formation. (**A**) Cell proliferation assays were carried out on MCF7 cells transfected with BCAS2 or empty vector (EV) and treated in the absence (EtOH) or presence of E2 for 8 days. Cell viability was assayed every 2 days, as described in materials and methods. BCAS2 overexpression caused a significant increase in proliferation both in the presence and absence of ligand (** *p* < 0.001) (**B**) Cell viability assays were carried out on MCF7 cells transfected with shBCAS2 or unrelated shE1 in the absence (EtOH) or presence of E2 and assayed after 96 h. Inhibition of BCAS2 caused a significant reduction in cell growth in the presence (* *p* < 0.05) of E2. (**C**) Colony formation of MCF7 cells transfected with BCAS2 or empty vector. 1000 transfected cells were plated per well and treated with E2 or TAM every 48 h for 12 days. Colonies were then stained and counted. Graphical representation of the number of colonies counted with respect to the number of cells plated is shown on right. Data represent three independent assays, * *p* < 0.05 compared to EV in the presence of EtOH and ** *p* < 0.001 compared to BCAS2 in the presence of EtOH.

In order to determine the effect that BCAS2 may have on breast cancer development, we used MCF7 breast cancer cells to determine its effect on proliferation and clonogenicity. Overexpression of BCAS2 caused a significant increase in estrogen dependent and independent cell proliferation (Figure 5A). Conversely, BCAS2 knockdown reduces cell viability in the presence of E2 compared to cells transfected with control shRNA (shE1) (Figure 5B).

We then carried out colony formation assays in MCF7 cells and observed a significant increase in clonogenicity upon BCAS2 overexpression and E2 treatment (Figure 5C).

### 2.7. BCAS2 Promotes Estradiol Dependent Cell Migration

**Figure 6:**
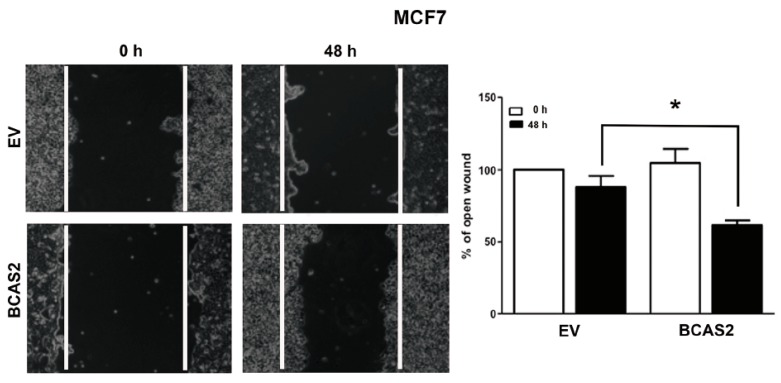
BCAS2 significantly increases cell migration. The effect of BCAS2 overexpression on MCF7 cell migration was determined using a wound healing assay. The wound healing rate of BCAS2 transfected cells was faster than empty vector (EV) in presence of E2. The open wound area at 0h was regarded as 100%. Values represent mean ± SD of three replicates. * *p* < 0.05 compared to the EV group at 48 h of E2 treatment. 10× magnification.

We analyzed the role of BCAS2 in cellular migration using wound-healing assays. Our data shows greater migration of cells overexpressing BCAS2 as compared to those transfected with EV after 48 h of E2 treatment (Figure 6).

### 2.8. Various Signal Transduction Pathways Are Involved in BCAS2 Activation 

**Figure 7:**
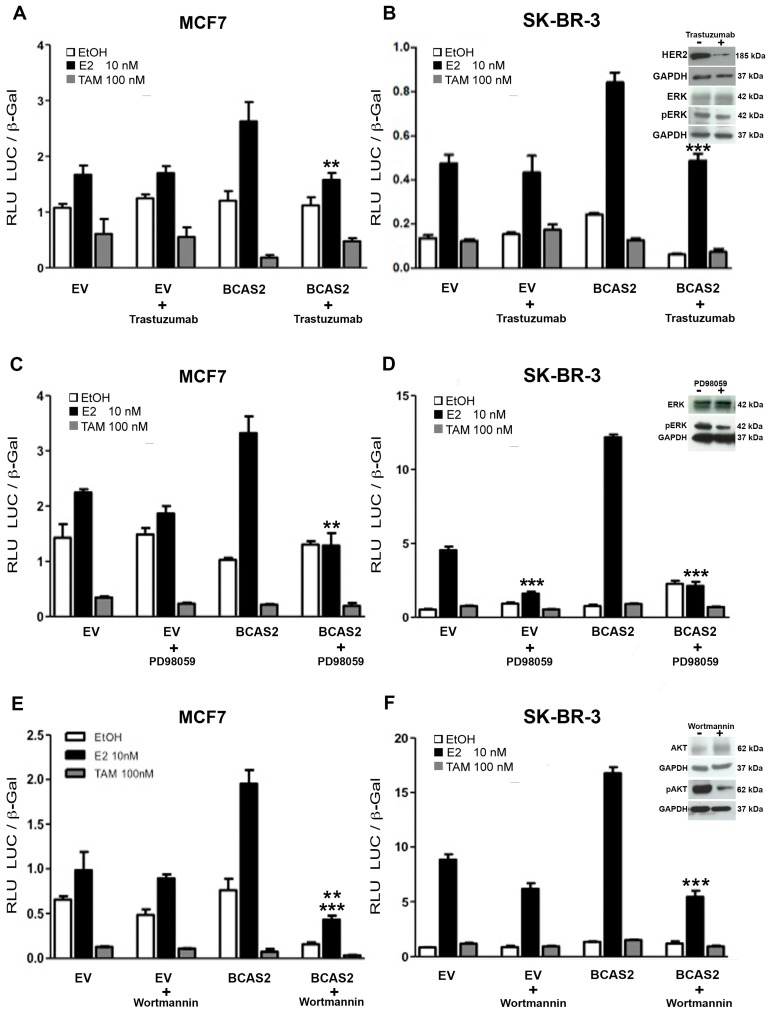
Signal transduction pathways involved in BCAS2 activation. (**A**, **C** and **E**) MCF7 cells were transiently transfected with an ERE-luciferase reporter vector (200 ng) and BCAS2 (100 ng) in the absence (EtOH) or presence of 10 nM E2 or 100 nM TAM along with 50 g of Trastuzumab or 10 mM of PD98059 or 100 nM of Wortmannin, as indicated, for 48h. Empty vector (EV) (100 ng) was used as negative control. (**B**, **D** and **F**) SK-BR-3 cells were transfected and treated in the same manner as MCF7 cells, except that 50 ng of pSG-ERα were also transfected. Relative luciferase activity (RLU LUC/β-gal) is shown as the mean ± SE of a representative assay carried out in triplicate, at least three times. Representative western blots show HER2 after 48 h of Trastuzumab treatment, ERK and ERK phosphorylation (pERK) after 48 h of Trastuzumab or PD98059 treatment, AKT and AKT phosphorylation (pAKT) after 48 h of Wortmannin treatment, each performed in triplicate or duplicate (Figure S2). GAPDH is used as control for protein loading. Statistical analysis was carried out using Student’s *t*-test; ** *p* < 0.005, *** *p* < 0.001.

It is well known that there is cross-talk between MAP kinase and/or PI3K/AKT signal transduction pathways and ERα in breast cancer cells, which promotes an increase in estrogen-mediated signaling and favors recruitment of coactivators, such as SRC-1 and SRC-3 [22,23]. Additionally, activation of these pathways by HER2, has been highly implicated in breast cancer and is known to promote endocrine resistance [24,25]. Therefore, we were interested in determining whether these signal transduction pathways were capable of impacting BCAS2 activation of ERα. We carried out transcriptional activation studies comparing two breast cancer cell lines using HER2, MEK-1 and PI3K inhibitors. MCF7 is an ERα positive cell line with low HER2 expression, while SK-BR-3 is ERα negative and overexpresses HER2 and thus, ERα was transfected. Our results show that the HER2 inhibitor, trastuzumab, has little effect on inhibiting ERα activation in our system. However, it causes complete abrogation of the BCAS2 induced increase in transcriptional activity in the presence of E2 (Figure 7A,B). The MEK-1 inhibitor, PD98059, further downstream in the MAPK signaling pathway, causes a more marked decrease in activity, inhibiting both ERα activity alone and the BCAS2 induced increase (Figure 7C). This effect is much more evident in SK-BR-3 cells, where ERα activity is almost completely abrogated (Figure 7D). This supports previous findings showing that MEK activation increases ER signaling [22] and thus suggests that this effect is probably caused by inhibiting ER and not as a direct effect on BCAS2. 

Conversely, blocking the PI3K/AKT (Wortmannin) signal transduction pathway shows little or no effect on ERα activity alone but shows an important effect on BCAS2 activity, inhibiting its ability to promote ERα transactivation (Figure 7E,F). 

## 3. Discussion

The identification and characterization of coregulators that modulate ERα activity is a crucial step in understanding the mechanisms that control, not only the expression of estrogen regulated genes but also the initiation and progression of breast cancer. In this study, we have reinforced and enhanced our knowledge about the role of BCAS2 as an ERα coactivator and its regulation in breast cancer cells. BCAS2 had previously been proposed as an ERα coactivator and had been shown to increase its transcriptional activity [14]. However, this group proposed that this activity was carried out solely through the AF-2 transcriptional activation domain. In this study, we confirmed direct interaction with the N-terminal domain of ERα in vitro, without finding interaction via de C-terminal domain (Figure 1B). Additionally, interaction with full-length ERα was observed both in the presence and absence of E2 and TAM (Figure 1C). The fact that there is interaction of BCAS with ERα in the presence of TAM further supports binding to the AF-1 domain, as the conformational change caused to the LBD does not abrogate BCAS2 binding. However, since there have been many sites known to harbor post transcriptional modifications involved in ERα activation in both the hinge and the LBD regions [26], we cannot rule out that some post translational modification is necessary for BCAS2 binding to the AF-2 region and that by using an in vitro GST-pulldown assay we are unable to observe this interaction. Additionally, our construct is lacking a small portion of the hinge region, so this may be a possible binding site for BCAS2. In this respect, there are a number of coactivator proteins that bind the hinge region, such as calmodulin, which promotes ERα dimerization [27].

Luciferase assays, carried out in breast cancer cell lines, confirmed that BCAS2 increases ERα transcriptional activity (Figure 2A,B). However, we also determined that BCAS2 increases transcription through both the AF-1 domain and the AF-2 domain, independently (Figure 2C,D). This is in contrast to data published by Qi et al. [14], who did not observe AF-1 activation by BCAS2 in CV-1 cells. It is likely that this cellular context does not support independent activation through the AF-1 domain alone while in HEPG2 cells both AFs have been found to function independently [16]. Thus, combined with our protein interaction data, this suggests that the effect on the AF-1 domain was carried out through its direct interaction with ERα while the positive effect on the AF-2 domain may require interaction with other coactivators that can interact directly with AF-2. This supports the idea that BCAS2 may be involved in promoting synergism between the AF-1 and AF-2 transactivation functions, as has been suggested for SRC-1 [16]. Thus, we tested the effect of BCAS2 on the activity of three coactivators that preferentially bind to AF-2 through their LxxLL sequences, although it has been shown that they can also bind to the N-terminal domain. We observed that the combination of BCAS2 with SRC-1, TIF2 or CBP increases the transcriptional activity of ERα compared to any of these coactivators alone (Figure 4). This implies that BCAS2 does not compete with these coactivators for receptor binding. This has been observed with other coactivators such as Tip60, which synergizes with TIF2 to increase ERβ transactivation [28]. Moreover, in MCF7 cells, the combination of BCAS2 with any of the coactivators tested, especially TIF2, also increases transcription in the presence of TAM and in some cases in the absence of ligand (Figure 4A). The combination of coactivators may be necessary to promote the agonist activity of TAM through AF-1, even though it abrogates their binding to AF-2. This is not consistently observed in the ER negative cell lines transfected with exogenous ERα (Figure 4C), suggesting that BCAS2 may play a role in TAM resistance in an ER+ cellular environment. Additionally, BCAS2 has been shown to be overexpressed in TAM resistant MCF7 cells and increased expression is associated to shorter relapse-free survival of breast cancer patients [29].

In order to ensure that BCAS2 is a bona fide coactivator, we demonstrated its existence on ER target promoter DNA and show that target gene expression is increased by BCAS2, at least for pS2, IGFBP2 and C3, both in the presence and absence of ligand (Figure 3). 

It is well known that the activity or expression of many ER coactivators increases during breast carcinogenesis and progression. However, the role that BCAS2 may play in cancer has been poorly elucidated. A number of studies have shown that it is amplified or overexpressed in breast tumors [29,30,31] and have suggested that this could influence the development of endocrine resistance, as has been observed for other coactivators [32,33,34,35]. Additionally, it has been found to be overexpressed in prostate cancer and to regulate the androgen receptor by increasing its mRNA expression and stabilizing AR protein [19]. Thus, we would expect that overexpression of BCAS2 can impact the physiologic environment of estrogen dependent breast cancer cells and be involved in carcinogenic processes. We studied the effect of overexpressing BCAS2 on cellular proliferation, clonogenicity and cell migration (Figure 5 and Figure 6). In all cases we observed that overexpression of BCAS2 enhances all of these processes. Additionally, this is also observed in the absence of ligand. This supports a role for BCAS2 in the progression of breast cancer, both before and after endocrine resistance sets in. Studies on other coactivators, such as SRC-1 have also shown that they have a role in increasing cell migration and proliferation in breast cancer [36,37], as well as increasing the expression of Twist and promoting metastasis [38]. The coactivators, CBP and p300, have also been shown to have the capacity to promote cell migration by increasing expression of metalloproteinases 2 and 9 [39]. Thus, these coactivators have been proposed as markers for breast cancer aggressiveness and malignancy. Our data showing that BCAS2 can synergize with these coactivators, as well as promote carcinogenic processes, shows it has important implications in breast cancer. Further studies need to be carried out to determine whether BCAS2 binds directly to other coactivators and to define its target interaction sequence on ERα. Additionally, assays that block these interactions, for example by using synthetic peptides [40], would greatly help to dissect its functions in ER transactivation, metastatic processes and endocrine resistance. In this regard, BCAS2 expression has also been shown to be upregulated by Estrogen Related Receptor β (ERRβ and both ERα and ERβ in the absence of E2, however addition of ligand inhibited its expression [31]. This work also showed that increased BCAS2 led to expression of MMP7 and cleavage of E-cadherin, supporting a role for BCAS2 in epithelial-mesenchymal transition. Conversely, BCAS2 seems to be involved in downregulating β-catenin resulting in a decrease of the pro-apoptotic protein FST. However, the inhibition of β-catenin observed after BCAS2 overexpression also derives in cyclin D1 downregulation, suggesting that proliferation may be inhibited when ERRβ is present. 

It has been previously demonstrated that there is cross-talk between ERα and various signal transduction pathways, including HER2, which is capable of activating both the MAPK and PI3K/AKT pathways [41]. HER2 overexpression has been observed in 20-30% of patients with breast cancer and has been implicated in TAM resistance, both acquired and de novo [42,43]. Additionally, various clinical studies have also indicated that HER2 overexpression is associated with a lower response to TAM treatment. Moreover, ectopic expression of HER2 in MCF7 cells is sufficient to confer resistance to TAM in these cells [44]. In our results, we show that inhibition of the MAPK and PI3K/AKT result in inhibition of ERα activity. However, the targets seem to be different. On one hand, blocking MEK-1 activity almost completely abrogates ERα activity (Figure 7C,D). On the other hand, inhibition of PI3K only inhibits the increase in activity promoted by BCAS2, without strongly affecting intrinsic ERα activity (Figure 7E,F). This highlights the importance of these signal transduction pathways in ERα regulation and the progression of estrogen-regulated breast cancer. The reduction in BCAS2 activity can probably be explained by a reduction in phosphorylation of both the receptor, as previously reported [25,45] and BCAS2. However, the direct effect on BCAS2 must still be determined.

## 4. Materials and Methods 

### 4.1. Yeast Two-Hybrid Screen

A yeast two-hybrid assay was carried out using the Matchmaker two-hybrid system 3 kit (Clontech Laboratories, Mountain View, CA, USA), following manufacturer’s instructions and as previously described [46]. Briefly, the N-terminal domain of ERα, amino acids (aa) 1-180, was used as bait to screen a human recurrent prostate carcinoma CWR-R1 cell line cDNA library. Positive clones were selected at high stringency in media lacking histidine, leucine, tryptophan and adenine, supplemented with 5 mM 3-Aminotriazole (3-AT) and X–Gal, to detect blue colonies. The resulting colonies were assayed for β-galactosidase activity. cDNAs from galactosidase positive yeast colonies were sequenced and analyzed using Basic Local Alignment Search Tool (BLAST) analysis. Full-length BCAS2 was recovered from the pACTII plasmid.

### 4.2. Cell Culture

All cell lines were obtained from ATCC (authenticated using STRS analysis) and tested for mycoplasma. Cell lines were cultured in media as suggested by ATCC, supplemented 5–10% fetal bovine serum (FBS) and antibiotics (100 U/mL penicillin and 100 mg/mL streptomycin) and incubated at 37 °C with 5% CO_2_ in a humidified atmosphere, for maintenance. The cell lines Hep-G2 and COS-7 were cultured in Eagle´s Minimum Essential Medium (EMEM) and Dulbecco’s Modified Eagle Medium (DMEM). Human breast cancer cell lines: MCF7, MDA-MB-231 and SK-BR-3, were cultured in EMEM with 0.01 mg/mL human recombinant insulin, Leibovitz’s L-15 Medium and McCoy’s 5a Medium, respectively. T-47D 3.17 (transfected to carry one stably integrated copy of a luciferase reporter gene driven by the MMTV promoter) [18] were cultured in RPMI 1640 with 0.2 units/mL bovine insulin. All media were supplemented with 5–10% fetal bovine serum (FBS) and antibiotics (100 U/mL penicillin and 100 mg/mL streptomycin) incubated at 37 °C with 5% CO_2_ in a humidified atmosphere, for maintenance. For studies with hormone treatments, phenol red-free media was used with charcoal-dextran treated serum (sFBS) to reduce the presence of hormones. 

### 4.3. Plasmids

Full-length BCAS2, as obtained in the yeast two-hybrid screen, was amplified from pACTII-BCAS2 and cloned into pcDNA3.1/His (Invitrogen, Carlsbad, CA, USA) to make pcDNA-BCAS2. All glutathione-S-transferase (GST) fusion constructs were generated in a pDEST15 vector, as previously described [42]. Estrogen and progesterone receptor vectors (pSG5-ERα and pSG5-PR) were donated by Dr. Pierre Chambon (INSERM, France). ER activation domain expression vectors, pERα-AF1 (aa 1-283) and pERα-AF2 (aa 144-595), as well as 3X-ERE-Luc were provided by Dr. Donald McDonnell (Duke University, USA) [17]. pSG5-SRC-1, pSG5-CBP and pSG5-TIF2 were obtained from Dr. Edwin Milgrom (INSERM, France).

### 4.4. Glutathione-S-Transferase (GST) Pull-Down Assays

GST fusion proteins: pGST-ERα Full (aa 1-595), pGST-ERα N (aa 1-180), pGST-ERα LBD (aa 264-595) were expressed in *E. coli* strain BL21, induced with 0.2% l-arabinose and purified with glutathione-Sepharose beads, according to manufacturer’s instructions (GE Healthcare). Biotin labeling and in vitro translation of BCAS2 was performed using the TNT^®^ T7 Quick Coupled Transcription/Translation System (Promega, Madison, WI, USA). 30 μL of bound GST fusion proteins was incubated with 10 μL of BCAS2 and incubated for 2 h in 500 μL of sonication buffer (20 mM Tris-HCl, pH 8, 100 mM NaCl, 0.7 mM EDTA, 0.05% Nonidet P-40, with protease inhibitors) in the presence or absence of 100 nM estradiol (E2). Beads were washed 5 times and eluted by boiling in 50 μL of 2× Laemmli loading buffer. Proteins were separated by SDS-PAGE followed by immunoblot using antibodies against GST (Santa Cruz Biotechnology, Dallas, TX, USA) and biotin (Promega, Madison, WI, USA) and chemiluminescent detection (GE Healthcare, Chicago, IL, USA). 

### 4.5. Coimmunoprecipitation

COS-7 cells (1.5 × 10^6^ cells per 10 cm dish) were transfected with 3μg pSG5-ERα and 3 μg pcDNA-BCAS2 using Lipofectamine 2000 (Invitrogen) for 5 h. Cells were then treated for 48 h in the absence (ethanol) or presence of 10 nM E2 and then harvested in lysis buffer (50 mM Tris-HCl, pH 8, 150 mM NaCl, 1% NP-40, 1 mM EDTA, 1 mM DTT). Cell debris was precipitated by centrifugation and the supernatant was incubated with protein A/G-Sepharose (Merck KGaA, Darmstadt, Germany) at 4 °C for 30 min to remove nonspecific interactions. The pre-cleared lysate was precipitated with antibodies against ERα (Santa Cruz Biotechnology) or BCAS2 (Abcam, Cambridge, UK) at 4 °C for 2 h and incubated overnight with protein A/G-Sepharose. After washing 4 times, the precipitates were eluted in loading buffer, separated by SDS-PAGE and transferred to a nitrocellulose membrane. Immunoblot was performed with anti-ERα and anti-BCAS2. Coimmunoprecipitation in MCF7 cells was carried out in the same manner, except incubations were carried out with EtOH, 10 nM E2 and 100 nM TAM.

### 4.6. Transient Transfection and Luciferase Assays

To determine the transcriptional activity effect of BCAS2 on SHRs, 7 × 10^4^ MCF7, T-47D 3.17 (T-47D-MTVL), SK-BR-3, MDA-MB-231 or Hep-G2 cells were seeded in 24-well plates and transfected with 200 ng of 3xERE-Luc or MMTV-Luc, 50 ng of receptor expressing plasmid, as required, 50 to 300 ng of the appropriate coregulator expression plasmid or empty vector (EV) and 10 ng of pCMV-β-galactosidase (Promega), as transfection control, for 6 h, using Lipofectamine 2000 (Invitrogen) as indicated by the manufacturer. Additionally, these experiments were performed using constructs expressing only the ERα AF-1 or ERα AF-2 in Hep-G2 cells, to determine whether the activity of BCAS2 requires one or both transcriptional activation sites. Cells were incubated for 48 h in the presence or absence of ligand. Ligands used for ERα were E2 or TAM and progesterone (P4) was used to activate the PR. To determine the effect of BCAS2 on the transcriptional activity of other ERα coactivators, 7 × 10^4^ MCF7, MDA-MB-231 and SK-BR-3 cells were transfected, as previously described, along with 50ng of SRC-1, CBP or TIF2. Finally, to determine the impact of HER2, PI3K and MEK-1 signaling pathways in BCAS2 activation, MCF7 and SK-BR-3 cells were transfected, as previously described, in presence of ethanol (EtOH), E2 or TAM along with Trastuzumab, PD98059 or Wortmannin for 48 h. Cell extracts were assayed for luciferase and β-galactosidase activities using a GloMax^®^ 96 microplate Luminometer (Promega). At least three independent transfections were performed in triplicate for each trial. Results are shown as relative light units (RLU) calculated as a ratio of luciferase and β-galactosidase activities.

### 4.7. Chromatin Immunoprecipitation (ChIP)

ChIP assays were performed as previously described [46]. Briefly, 3 × 10^6^ MCF7 cells were grown in 10 cm dishes in phenol red-free medium with 2% sFBS for 2 days before treatment with vehicle (EtOH) or 10 nM E2 for 30 min – 2 h, as indicated. Cells were crosslinked and harvested in cold PBS and protease inhibitor cocktail (Roche) plus 1mM DTT. The cell button was processed using a ChIP Kit (Upstate) following the manufacturer’s instructions. Immunoprecipitation was performed overnight at 4 °C with the addition of 3 g normal rabbit IgG, rabbit anti-ERα (Santa Cruz Biotechnology), mouse monoclonal anti-BCAS2 (Abcam) or methyl histone H3 antibody (Upstate Biotechnology, Lake Placid, NY, USA). Real-time PCR was carried out to amplify promoter regions from the pS2 (TFF1) [20] and WISP2 [21] genes. Oligonucleotides used were: pS2: fwd 5′-CCATGTTGGCCAGGCTAGTC-3′, rev 5′-ACAACAGTGGCTCACGGGCT-3′; WISP2: fwd 5′-TGTTGTGCCTCCAGCTCCTG-3′, rev 5′-GGTTTCTGGCAGGCAGATT-3′.

### 4.8. Real-Time Quantitative PCR

Real-time PCR was carried out using the SYBR Green PCR Master Mix (Applied Biosystems, Foster City, CA, USA) according to the manufacturer’s protocol, using a LightCycler 480 (Roche Indianapolis, IN, USA).

### 4.9. Expression of Endogenous Genes

To monitor endogenous expression of estrogen-responsive genes, we used MCF7 cells transfected, as described above, with pcDNA-BCAS2 and treated with E2 for 24 h. The effect of overexpression of BCAS2 on the endogenous expression of E2-dependent genes was determined by RTqPCR. Total RNA was isolated using TRIzol (Invitrogen), following manufacturer’s instructions. We measured mRNA levels of pS2, C3, IGFBP2 and c-Myc. The cDNA was obtained from 100 ng of total RNA using SuperScript Vilo cDNA synthesis (Invitrogen, Carlsbad, CA, USA). Glyceraldehyde-3-phosphate dehydrogenase (human GAPDH) mRNA amplification was used for assay normalization and relative units are reported. Oligonucleotides used were: pS2 fwd 5′-CACCATGGAGAACAAGGTGA-3′, rev 5′-TGACACCAGGAAAACCACAA-3′; C3: fwd 5′-ACCAGCAGACCGTAACCATC-3′, rev 5´-GCAGCCTTGACTTCCACTTC-3′; IGFBP2: fwd 5′-CCTCAAGTCGGGTATGAAGG-3′, rev 5′-ACCTGGTCCAGTTCCTGTTG-3′; c-MYC: fwd 5′-AGAGAAGCTGGCCTCCTACC-3′, rev 5′-CGTCGAGGAGAGCAGAGAAT-3′; GAPDH: fwd 5′-CCTCAACGACCACTTTGTCA-3’; rev: 5’-CCCTGTTGCTGTAGCCAAAT-3’. The data shown were performed three times in triplicate. 

### 4.10. shRNA Transfection

Lentivirus stocks were produced in HEK 293T cells by transfection with pLKO.1-BCAS2 siRNA constructs or control siRNA (shRNAs; Sigma Aldrich), packaging plasmid (pCMV R8.91) and envelope plasmid (pVSVG) using Calcium phosphate. Viruses were harvested and concentrated as previously described [42] 3 × 10^5^ MCF7 cells were infected by spin inoculation system [47] centrifuging the virus particles with the cells at 1200 rpm for 2h at room temperature. Cells infected with vectors expressing shRNA-BCAS2 or shRNA-E1 (negative control) pLKO.1 and were selected with 2 g/mL of puromycin (Sigma-Aldrich) 24 h after infection.

### 4.11. Cell Viability Assays

Cell viability was assessed using a tetrazolium salt WST-1 assay (Quick Cell Proliferation, BioVision, Milpitas CA, USA). MCF7 cells (5 × 10^5^) were seeded in 6-well plates and transfected with 3 μg pcDNA-BCAS2 or EV, as described previously and left for 48 h. Cells were then seeded in 96-well plates and treated in the absence (EtOH) or presence of E2 and incubated for up to 8 days. Cell viability measurements were taken every 48 h. After incubation, 10 μL of 5 mg/mL WST was added to the cells and the reaction was incubated for 3 h at 37 °C. The assay was read at an absorbance of 450 nm using a microplate reader (BioTek Synergy H1 Hybrid Multi-Mode Microplate Reader) and survival of cells was calculated by dividing the absorbance of the transfected cells by that of the non-transfected. All tests were performed in quadruplicate. Additionally, cell viability was assayed in the same manner after transfecting BCAS2 shRNA constructs, as described previously. Cells were seeded in 96 well plates and allowed to grow for 96 h before determining cell viability. All tests were performed in quadruplicate.

### 4.12. Colony Formation and Wound Healing Assays

To perform clonogenicity assays, MCF7 cells were BCAS2 or EV transfected and 1000 cells/well were seeded in 6-well plates. Treatments were carried out with E2, TAM or vehicle (EtOH) for 12 days. Cells were then dyed with crystal violet 0.05% and the number of colonies was counted. Colony numbers were assessed visually and colonies containing >50 normal-appearing cells were counted. Pictures were taken using a digital camera. Number of colonies is reported as % of colonies (PE), where PE = (# colonies formed/# cells plated) × 100 [48]. Experiments were performed three times. 

To assess cell motility, wound healing assays were carried out. MCF7 cells were BCAS2 or EV transfected and 500,000 cells/well were plated in 6-well plates. Once cells were 90% confluent, the surface was scratched with a 200 μL tip and cells were treated with E2 for 48 h. Experiments were carried out in the presence of a proliferation inhibitor, AraC (20nM), for 48 h. Pictures were taken every 24 h for 48 h and the % of open wound was calculated. 

### 4.13. Statistical Analysis

All experiments were repeated at least three times and data are expressed as the mean ± standard deviation. Statistical analysis of the proliferation results was carried out by two-way ANOVA followed by Bonferroni post tests using Graph Pad Prism 6.0 for Mac. Statistical analysis for luciferase assays was carried out using Student´s *t*-test or one-way ANOVA followed by Dunnet port test.

## 5. Conclusions

We have shown that BCAS2 is a bona fide coactivator of ERα in breast cancer cell lines, being recruited to target gene promoters and increasing expression of a subset of E2 regulated genes. BCAS2 functions primarily through its interaction with AF-1 and collaborates with AF-2 coactivators, having a role in both independent and synergistic activity of these two transcriptional activation functions. Additionally, BCAS2 is involved in promoting various processes of carcinogenesis both in the presence and absence of estradiol. This suggests a role for BCAS2 overexpression in promoting ERα activity in a reduced estrogen environment, as is found during hormonal treatment for breast cancer. Finally, BCAS2 is regulated by the PI3K/AKT signaling pathway. However, the mechanisms involved in this regulation must be studied further.

## Supplementary Materials

Supplementary materials can be found at https://www.mdpi.com/1422-0067/20/4/966/s1. Supplementary Figure S1. Coimmunoprecipitation assays in MCF7 cells in the presence of ethanol (OH), E_2_ (10nM) or TAM (100nM) showing interaction in the presence or absence of ligand. Input refers to pre-clear protein extracts. Supplementary Figure S2. Raw western blot data studies using inhibitors of signal transduction pathways. (**A**) Three independent assays showing HER2 expression in SK-BR-3 cells in the presence or absence of the HER 2 inhibitor trastuzumab. (**B**) Two independent assays showing AKT and pAKT expression in MCF7 cells in the presence and absence of Trastuzumab and AKT activation inhibitor wortmannin. (**C**) Two independent assays showing ERK and pERK expression in MCF7 cells in the presence or absence of MEK inhibitor PD98059.

## Abbreviations

ERα	Estrogen Receptor alpha
BCAS2	Breast Cancer Amplified Sequence 2
TAM	Tamoxifen
E2	Estradiol
P4	Progesterone
EtOH	Ethanol
aa	amino acids
GAPDH	Glyceraldehyde-3-phosphate dehydrogenase
SRC-1	Steroid Receptor coactivator-1
TIF2	Transcriptional Intermediary Factor 2
CBP	CREB Binding Protein

## References

[1] Sommer S., Fuqua S.A. (2001). Estrogen receptor and breast cancer. Semin. Cancer Biol..

[2] Bender L.M., Nahta R. (2008). Her2 cross talk and therapeutic resistance in breast cancer. Front. Biosci..

[3] Hurvitz S.A., Pietras R.J. (2008). Rational management of endocrine resistance in breast cancer: A comprehensive review of estrogen receptor biology, treatment options and future directions. Cancer.

[4] Santen R., Cavalieri E., Rogan E., Russo J., Guttenplan J., Ingle J., Yue W. (2009). Estrogen mediation of breast tumor formation involves estrogen receptor-dependent, as well as independent, genotoxic effects. Ann. N. Y. Acad. Sci..

[5] Robinson D.R., Wu Y.M., Vats P., Su F., Lonigro R.J., Cao X., Kalyana-Sundaram S., Wang R., Ning Y., Hodges L. (2013). Activating ESR1 mutations in hormone-resistant metastatic breast cancer. Nat. Genet..

[6] Thomas C., Gustafsson J.Å. (2015). Estrogen receptor mutations and functional consequences for breast cancer. Trends Endocrinol. Metab..

[7] Glass C.K., Rosenfeld M.G. (2000). The coregulator exchange in transcriptional functions of nuclear receptors. Genes Dev..

[8] McKenna N.J., O’Malley B.W. (2002). Combinatorial Control of Gene Expression by Nuclear Receptors and Coregulators. Cell.

[9] Heery D.M., Kalkhoven E., Hoare S., Parker M.G. (1997). A signature motif in transcriptional co-activators mediates binding to nuclear receptors. Nature.

[10] Torchia J., Glass C., Rosenfeld M.G. (1998). Co-activators and co-repressors in the integration of transcriptional responses. Curr. Opin. Cell Biol..

[11] Xu J., Wu R.C., O’Malley B.W. (2009). Normal and cancer-related functions of the p160 steroid receptor co-activator (SRC) family. Nat. Rev. Cancer.

[12] Perissi V., Jepsen K., Glass C.K., Rosenfeld M.G. (2010). Deconstructing repression: Evolving models of co-repressor action. Nat. Rev. Genet..

[13] Xu L., Glass C.K., Rosenfeld M.G. (1999). Coactivator and corepressor complexes in nuclear receptor function. Curr. Opin. Genet. Dev..

[14] Qi C., Zhu Y.T., Chang J., Yeldandi A.V., Rao M.S., Zhu Y.J. (2005). Potentiation of estrogen receptor transcriptional activity by breast cancer amplified sequence 2. Biochem. Biophys. Res. Commun..

[15] Kuo P.C., Tsao Y.P., Chang H.W., Chen P.H., Huang C.W., Lin S.T., Weng Y.T., Tsai T.C., Shieh S.Y., Chen S.L. (2009). Breast cancer amplified sequence 2, a novel negative regulator of the p53 tumor suppressor. Cancer Res..

[16] Tzukerman M.T., Esty A., Santiso-Mere D., Danielian P., Parker M.G., Stein R.B., Pike J.W., McDonnell D.P. (1994). Human estrogen receptor transactivational capacity is determined by both cellular and promoter context and mediated by two functionally distinct intramolecular regions. Mol. Endocrinol..

[17] Métivier R., Penot G., Flouriot G., Pakdel F. (2001). Synergism between ERalpha transactivation function 1 (AF-1) and AF-2 mediated by steroid receptor coactivator protein-1: Requirement for the AF-1 alpha-helical core and for a direct interaction between the N- and C-terminal domains. Mol. Endocrinol..

[18] Truss M., Bartsch J., Schelbert A., Hache R.J., Beato M. (1995). Hormone induces binding of receptors and transcription factors to a rearranged nucleosome on the MMTV promoter in vivo. EMBO J..

[19] Kuo P.C., Huang C.W., Lee C.I., Chang H.W., Hsieh S.W., Chung Y.P., Lee M.S., Huang C.S., Tsao L.P., Tsao Y.P. (2015). BCAS2 promotes prostate cancer cells proliferation by enhancing AR mRNA transcription and protein stability. Br. J. Cancer.

[20] Merrell K.W., Crofts J.D., Smith R.L., Sin J.H., Kmetzsch K.E., Merrell A., Miguel R.O., Candelaria N.R., Lin C.Y. (2011). Differential recruitment of nuclear receptor coregulators in ligand-dependent transcriptional repression by estrogen receptor-α. Oncogene.

[21] Fritah A., Redeuilh G., Sabbah M. (2006). Molecular cloning and characterization of the human WISP-2/CCN5 gene promoter reveal its upregulation by oestrogens. J. Endocrinol..

[22] Atanaskova N., Keshamouni V.G., Krueger J.S., Schwartz J.A., Miller F., Reddy K.B. (2002). MAP kinase/estrogen receptor cross-talk enhances estrogen-mediated signaling and tumor growth but does not confer tamoxifen resistance. Oncogene.

[23] Wu V.S., Kanaya N., Lo C., Mortimer J., Chen S. (2015). From bench to bedside: What do we know about hormone receptor-positive and human epidermal growth factor receptor 2-positive breast cancer?. J. Steroid Biochem. Mol. Biol..

[24] Takimoto G.S., Graham J.D., Jackson T.A., Tung L., Powell R.L., Horwitz L.D., Horwitz K.B. (1999). Tamoxifen resistant breast cancer: Coregulators determine the direction of transcription by antagonist-occupied steroid receptors. J. Steroid Biochem. Mol. Biol..

[25] Osborne C.K., Shou J., Massarweh S., Schiff R. (2005). Crosstalk between estrogen receptor and growth factor receptor pathways as a cause for endocrine therapy resistance in breast cancer. Clin. Cancer Res..

[26] Le Romancer M., Poulard C., Cohen P., Sentis S., Renoir J.-M., Corbo L. (2011). Cracking the Estrogen Receptor’s Posttranslational Code in Breast Tumors. Endoc. Rev..

[27] Li Z., Zhang Y., Hedman A.C., Ames J.B., Sacks D.B. (2017). Calmodulin Lobes Facilitate Dimerization and Activation of Estrogen Receptor-α. J. Biol. Chem..

[28] Lee M.T., Leung Y.K., Chung I., Tarapore P., Ho S.M. (2013). Estrogen Receptor b (ERb1) Transactivation Is Differentially Modulated by the Transcriptional Coregulator Tip60 in a cis-Acting Element-dependent Manner. J. Biol. Chem..

[29] Nagasaki K., Maass N., Manabe T., Hanzawa H., Tsukada T., Kikuchi K., Yamaguchi K. (1999). Identification of a novel gene, DAM1, amplified at chromosome 1p13.3-21 region in human breast cancer cell lines. Cancer Lett..

[30] Worsham M.J., Pals G., Schouten J.P., Miller F., Tiwari N., van Spaendonk R., Wolman S.R. (2006). High-resolution mapping of molecular events associated with immortalization, transformation and progression to breast cancer in the MCF10 model. Breast Cancer Res. Treat..

[31] Sengupta D., Bhargava D.K., Dixit A., Sahoo B.S., Biswas S., Biswas G., Mishra S.K. (2014). ERRβ signaling through FST and BCAS2 inhibits cellular proliferation in breast cancer cells. Br. J. Cancer.

[32] Schiff R., Massarweh S., Shou J., Osborne C.K. (2003). Breast cancer endocrine resistance: How growth factor signaling and estrogen receptor coregulators modulate response. Clin. Cancer Res..

[33] Zhao W., Zhang Q., Kang X., Jin S., Lou C. (2009). AIB1 is required for the acquisition of epithelial growth factor receptor-mediated tamoxifen resistance in breast cancer cells. Biochem. Biophys. Res. Commun..

[34] Nagalingam A., Tighiouart M., Ryden L., Joseph L., Landberg G., Saxena N.K., Sharma D. (2012). Med1 plays a critical role in the development of tamoxifen resistance. Carcinogenesis.

[35] Redmond A.M., Byrne C., Bane F.T., Brown G.D., Tibbitts P., O’Brien K., Hill A.D., Carroll J.S., Young L.S. (2015). Genomic interaction between ER and HMGB2 identifies DDX18 as a novel driver of endocrine resistance in breast cancer cells. Oncogene.

[36] Qin L., Chen X., Wu Y., Feng Z., He T., Wang L., Liao L., Xu J. (2011). Steroid receptor coactivator-1 upregulates integrin α_5_ expression to promote breast cancer cell adhesion and migration. Cancer Res..

[37] Walsh C.A., Qin L., Tien J.C., Young L.S., Xu J. (2012). The function of steroid receptor coactivator-1 in normal tissues and cancer. Int. J. Biol. Sci..

[38] Qin L., Liu Z., Chen H., Xu J. (2009). The steroid receptor coactivator-1 regulates twist expression and promotes breast cancer metastasis. Cancer Res..

[39] Santer F.R., Höschele P.S., OH S.J., Erb H.H., Bouchal J., Cavarretta I.T., Parson W., Meyers D.J., Cole P.A., Culig Z. (2011). Inhibition of the Acetyltransferases p300 and CBP Reveals a Targetable Function for p300 in the Survival and Invasion Pathways of Prostate Cancer Cell Lines. Mol. Cancer Ther..

[40] Leclercq G., Gallo D., Cossy J., Laïos I., Larsimont D., Laurent G., Jacquot Y. (2011). Peptides Targeting Estrogen Receptor Alpha-Potential Applications for Breast Cancer Treatment. Curr. Pharm. Des..

[41] Shou J., Massarweh S., Osborne C.K., Wakeling A.E., Ali S., Weiss H., Schiff R. (2004). Mechanisms of tamoxifen resistance: Increased estrogen receptor-HER2/neu cross-talk in ER/HER2-positive breast cancer. J. Natl. Cancer Inst..

[42] Kurokawa H., Lenferink A.E., Simpson J.F., Pisacane P.I., Sliwkowski M.X., Forbes J.T., Arteaga C.L. (2000). Inhibition of HER2/neu (erbB-2) and mitogen-activated protein kinases enhances tamoxifen action against HER2-over-expressing, tamoxifen-resistant breast cancer cells. Cancer Res..

[43] Arpino G., Green S.J., Allred D.C., Lew D., Martino S., Osborne C.K., Elledge R.M. (2004). HER-2 amplification, HER-1 expression and tamoxifen response in estrogen receptor-positive metastatic breast cancer: A southwest oncology group study. Clin. Cancer Res..

[44] Benz C.C., Scott G.K., Sarup J.C., Johnson R.M., Tripathy D., Coronado E., Shepard H.M., Osborne C.K. (1992). Estrogen-dependent, tamoxifen-resistant tumorigenic growth of MCF7 cells transfected with HER2/neu. Breast Cancer Res. Treat..

[45] Osborne C.K., Bardou V., Hopp T.A., Chamness G.C., Hilsenbeck S.G., Fuqua S.A., Wong J., Allred D.C., Clark G.M., Schiff R. (2003). Role of the estrogen receptor coactivator AIB1 (SRC-3) and HER-2/neu in tamoxifen resistance in breast cancer. J. Natl. Cancer Inst..

[46] Noriega-Reyes M.Y., Rivas-Torres M.A., Oñate-Ocaña L.F., Vallés A.J., Baranda-Avila N., Langley E. (2015). Novel role for PINX1 as a coregulator of nuclear hormone receptors. Mol. Cell. Endocrinol..

[47] Wiznerowicz M., Trono D. (2003). Conditional suppression of cellular genes: Lentivirus vector-mediated drug-inducible RNA interference. J. Virol..

[48] Franken N.A., Rodermond H.M., Stap J., Haveman J., Van Bree C. (2006). Clonogenic assay of cells in vitro. Nat. Protoc..

