# Supplementary material for: BCAS2 Enhances Carcinogenic Effects of Estrogen Receptor Alpha in Breast Cancer Cells
Source: Int J Mol Sci. 2019 Feb 22;20(4):966. doi: 10.3390/ijms20040966 (PMC6412365; doi:10.3390/ijms20040966)
Supplement: Supplementary file 1 [file ijms-20-00966-s001.pdf]

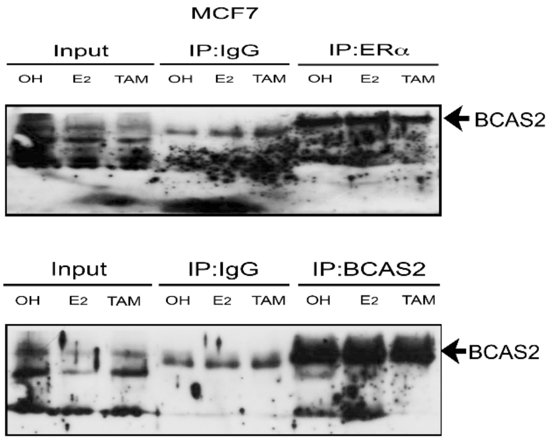

Supplementary Figure S1. Coimmunoprecipitation assays in MCF7 cells in the presence of ethanol (OH), E_2 (10nM) or TAM (100nM) showing interaction in the presence or absence of ligand. Input refers to pre-clear protein extracts.

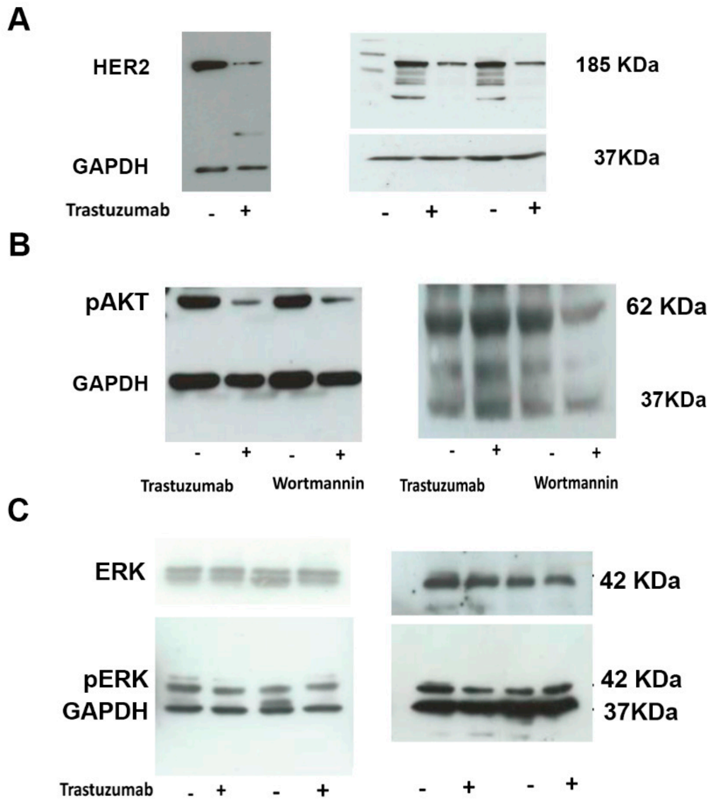

Supplementary Figure S2. Raw western blot data studies using inhibitors of signal transduction pathways. (**A**) Three independent assays showing HER2 expression in SK-BR-3 cells in the presence or absence of the HER 2 inhibitor trastuzumab. (**B**) Two independent assays showing AKT and pAKT expression in MCF7 cells in the presence and absence of Trastuzumab and AKT activation inhibitor wortmannin. (**C**) Two independent assays showing ERK and pERK expression in MCF7 cells in the presence or absence of MEK inhibitor PD98059.
